# Metabolic responses of plankton to warming during different productive seasons in coastal Mediterranean waters revealed by in situ mesocosm experiments

**DOI:** 10.1038/s41598-022-12744-x

**Published:** 2022-05-30

**Authors:** Tanguy Soulié, Francesca Vidussi, Justine Courboulès, Sébastien Mas, Behzad Mostajir

**Affiliations:** 1 MARBEC (MARine Biodiversity, Exploitation and Conservation), Univ Montpellier, CNRS, Ifremer, IRD, Montpellier, France; 2 MEDIMEER (MEDIterranean platform for Marine Ecosystems Experimental Research), OSU OREME, CNRS, Univ Montpellier, IRD, INRAE, Sète, France

**Keywords:** Ecology, Environmental sciences, Limnology, Ocean sciences

## Abstract

The response of coastal lagoon plankton communities to warming was studied during two in situ mesocosm experiments in spring and fall of 2018 in the Mediterranean. Phytoplankton biomass, gross primary production (GPP), community respiration (R), phytoplankton growth (µ), and loss (*l*) rates were estimated using high-frequency chlorophyll-*a* fluorescence and dissolved oxygen sensors, and daily sampling was used to evaluate the nutrient status and phytoplankton pigment functional groups. Warming strongly depressed the dominant phytoplankton functional groups, mainly the prymnesiophytes, diatoms (spring), and green flagellates (fall). It favored minor groups such as the dinoflagellates (spring) and diatoms (fall). In spring, warming depressed GPP and R by half; however, µ (+ 18%) and *l* (+ 37%) were enhanced. In contrast, both GPP and µ were enhanced by 21% and 28%, respectively, in fall, and no effects were observed for R and *l*. Warming strongly decreased phytoplankton biomass and oxygen production in spring, and enhanced them, to a lesser extent, in fall. This led to an overall loss of production over both seasons. This study improves understanding of the contrasting effects of warming during two productive seasons, which depend on plankton community composition and interactions between components and environmental conditions.

## Introduction

Surface ocean temperatures have increased globally in recent decades and are expected to have considerable effects on plankton communities and their metabolic processes^1,2^. According to Arrhenius’ Law^3^, an increase in temperature directly enhances plankton metabolic rates through interactions with biological processes and enzymatic activities^4,5^. The composition of plankton communities can also be affected, as more thermally adapted groups outcompete others. Thus, increases in water temperature affect plankton food webs, as they modify the interactions between organisms^6,7^ and lead to complex trophic cascades^8^. Warming can also increase the abundance and activity of grazers^9–11^, resulting in potentially higher grazing pressures on primary and secondary producers. Warming can also modify the physical parameters of the water column by altering resource availability and plankton metabolism.

Global-scale warming is not homogenous, as certain regions are more sensitive than others. Among these hotspots, the Mediterranean Sea is considered particularly sensitive to global warming^12,13^. The sea surface temperature (SST) here is predicted to increase by 3 °C by the end of the century^14^, and it will have important consequences for the plankton community, especially during highly productive spring blooms^8,15^. However, little is known about the effects of warming during fall, which is the other productive season^7^. It is unclear whether warming will have similar effects on different plankton communities and environmental conditions.

Plankton communities are major contributors to multiple biogeochemical cycles, notably those of carbon and oxygen^16^. Phytoplankton produces oxygen through gross primary production (GPP), and all planktonic organisms consume oxygen through aerobic respiration (R). Consequently, plankton plays an important role in the capacity of marine ecosystems to act as net sources or sinks of oxygen^17^. In a natural environment, phytoplankton growth (µ) and losses (*l*) due to grazing, viral lysis, natural mortality, and sedimentation are the main factors driving the dynamics of phytoplankton and, therefore, primary production^18^. The estimation of growth and loss rates is of prime interest for assessing the response of phytoplankton to experimental warming, and predicting the effects of global warming on primary production on a broader scale.

Traditional methods for estimating plankton processes, such as dilution experiments^19^ for µ and *l*, or bottle incubations for GPP and R^20^, are often time-consuming, labor-intensive, and impractical for in situ experimentation in remote locations, as they rely on manual sampling. Conversely, recent developments that allow the use of high-frequency automated sensors to measure dissolved oxygen and chlorophyll-*a* (chl-*a*) fluorescence (a proxy of phytoplankton biomass), and their fluctuations over time, offer several advantages in the estimation of GPP, R, µ, and *l*, in mesocosm experiments^21^. Long-term datasets can be obtained in a non-invasive manner, even if the mesocosms are not easily accessible. By reducing measurement intervals down to a minute, sensors offer higher resolutions and larger amounts of data than manual sampling^22^, which increases statistical robustness when testing for the effects of perturbations.

Employing recent developments in the application of high-frequency automated sensors, we conducted two in situ mesocosm experiments during two productive seasons (spring and fall of 2018). The responses of natural coastal Mediterranean plankton communities to warming were compared in the Thau Lagoon, located in the northwestern Mediterranean. In this study, during both in situ mesocosm experiments, triplicate in situ mesocosms were heated at + 3 °C above the ambient lagoon temperature, and compared to other mesocosms, in triplicate, kept at lagoon temperature. High-frequency sensor monitoring was used to evaluate the response of GPP, R, µ, and *l* to warming, and was combined with daily manual sampling to identify potential environmental drivers (i.e., nutrient availability) and the major phytoplankton contributors (i.e., community composition as assessed through pigment biomarkers).

## Results

### Effect of warming on physical and chemical conditions

**Figure 1:**
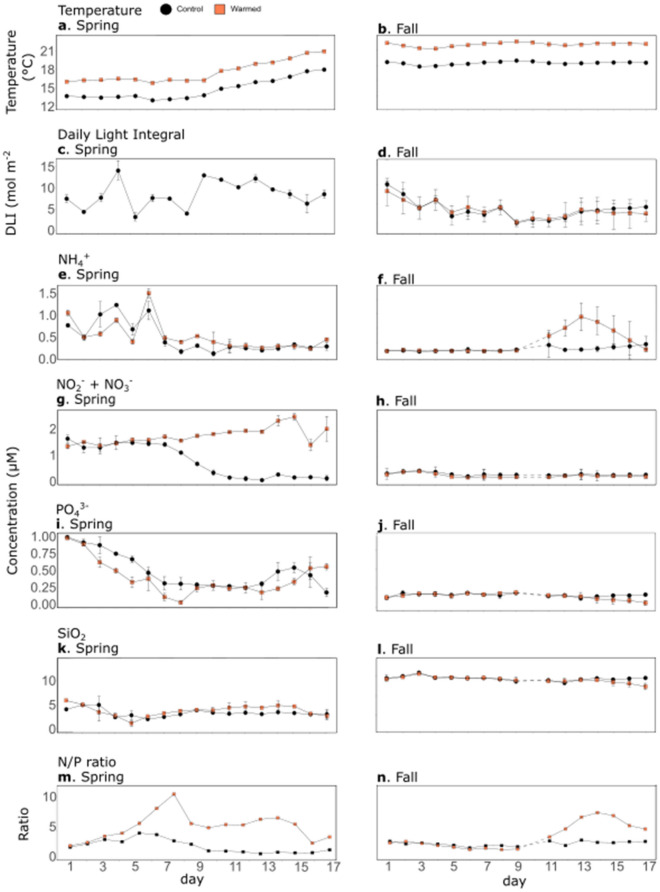
Time series of physical and chemical variables. Water temperature (**a**, **b**), Daily Light Integral (DLI, **c**, **d**), ammonium (NH_4_^+^, **e**, **f**), nitrates (NO_3_^−^ + NO_2_^−^, **g**, **h**), orthophosphate (PO_4_^3−^, **i**, **j**), silicate (SiO_2_, **k**, **l**) concentrations, and N/P ratio (**m**, **n**) over the course of the spring (**a**, **c**,**e**, **g**, **i**, **k**, **m**) and fall (**b**, **d**, **f**, **h**, **j**, **l**, **n**) experiments in the control (black) and the warmed (orange) treatments. Error bars represent range of observation for the two mesocosms per treatment in spring and the standard deviation for the three mesocosms per treatment in fall. Dotted lines represent the missing data on d10 of the fall experiment due to bad weather conditions. Due to technical difficulties, DLI could not be calculated in the warmed mesocosms of the spring experiment.

The water temperature in the warmed treatment was increased by 2.87 ± 0.20 °C in spring and 3.04 ± 0.08 °C in fall, compared to the control (Fig. 1a,b, Table 1). The average temperature in the control treatment, throughout the duration of the experiment, was about 4 °C cooler in spring (14.84 ± 0.03 °C) than in fall (19.01 ± 0.02 °C). In spring, the temperature naturally increased by approximately 4.19 °C from day (d) 10, until the end of the experiment, whereas it remained relatively constant in the fall experiment. It displayed higher diurnal variations in spring than in fall: over the course of the experiments, daily temperature variation ranged from 0.87 to 1.98 °C in spring and from 0.23 to 1.12 °C in fall. The average Daily Light Integral (DLI) in the control treatment, was almost twice as high in the spring experiment (7.93 ± 0.61 mol m^−2^ d^−1^) than during the fall (4.61 ± 0.52 mol m^−2^ d^−1^) (Fig. 1c,d). Warming did not significantly alter the DLI in fall (Table 1); however, the DLI could not be measured in the warm mesocosms in spring, owing to technical problems.

**Table 1:** Summary of the *p* values obtained by Repeated Measures Analyses Of VAriance (RM-ANOVA, with treatment as fixed factor and time as random factor) comparing physical parameters and nutrient concentrations in the warmed and control mesocosms.

Experiment	Variable	Period	*P* value	Cohen’s *d*
Spring	Temperature	2–17	**< 1.0 × 10**^−**4**^ (F_1,16_ = 3180)	**1.62**
	DLI	*n.d*	*n.d*	*n.d*
	NH_4_^+^	2–17	0.29 (KW)	0.07
	NO_3_^−^ + NO_2_^−^	2–17	**2.1** × **10**^−**5**^ (KW)	**2.06**
		8–17	**1.6** × **10**^−**5**^ (KW)	**4.91**
	PO_4_^3−^	2–17	**0.04** (F_1,14_ = 5.1)	–0.47
		2–10	**4.4** × **10**^−**3**^ (F_1,8_ = 15.3)	–0.62
	SiO_2_	2–17	0.19 (F_1,14_ = 1.9)	0.36
		10–17	**0.03** (KW)	**1.53**
	N/P ratio	2–17	**1.0 × 10**^−**4**^ (F_1,16_ = 32.76)	**1.62**
Fall	Temperature	1–17	**1.8** × **10**^−**7**^ (KW)	**4.66**
	DLI	1–17	0.79 (F_1,15_ = 0.71)	–0.07
	NH4 +	1–17	0.55 (KW)	0.78
		11–17	**0.01** (KW)	**1.93**
	NO_3_^−^ + NO_2_^−^	1–17	**8.0** × **10**^−**5**^ (KW)	–**0.82**
	PO_4_^3−^	1–17	0.07 (KW)	–0.47
		15–17	**0.04** (KW)	–**5.18**
	SiO_2_	1–17	**0.02** (F_1,15_ = 3.1)	–0.42
		15–17	**4** × **10**^−**3**^ (KW)	–**3.21**
	N/P ratio	1–17	**0.02** (F_1,15_ = 6.3)	**0.83**
		11–17	**1.6 × 10**^−**3**^ (F_1,6_ = 29.8)	2.9

A Kruskal–Wallis test, indicated by “KW”, was used when the assumptions for a parametric test were not met, despite transforming the data. *P* < 0.05 was considered statistically significant (values indicated in bold). Large and very large Cohen’s *d* effect sizes are also indicated in bold. Tests were performed from d2 of the spring experiment, when warming reached + 3 °C. (*n.d*. not determined, *DLI* Daily Light Integral).

Nutrient concentrations were measured daily in all mesocosms (Fig. 1, Table 1). Ammonium (NH_4_^+^) concentrations were higher in spring than in fall in the controls (0.45 ± 0.08 µM, and 0.41 ± 0.05 µM, respectively). Ammonium concentrations were significantly different, between the control and warmed mesocosms, only at the end of the fall experiment (warmed with a mean of 0.58 ± 0.23 µM, between d11–17; and control with a mean of 0.21 ± 0.09 µM, between d11–17). In contrast, ammonium concentrations did not vary between the control and warmed mesocosms in spring (Table 1). Cohen’s effect size (*d*) was used to evaluate the magnitude of the effect of warming. Regarding ammonium concentrations, the values were ten times larger in spring than in fall.

The nitrate + nitrite (NO_3_^−^ + NO_2_^−^) concentrations in the control treatments were higher in the spring, compared to the fall experiment (0.71 ± 0.08 µM and 0.23 ± 0.02 µM, respectively). Moreover, warming had different effects, depending on the experiment. In spring, the nitrate + nitrite concentrations were significantly higher in the warmed mesocosms from d8 until the end of the experiment, with an average difference of 540.5% between the warmed and control mesocosms, corresponding to a very large *d*. In fall, nitrate + nitrite concentrations were significantly lower in the warmed mesocosms than in the control, with an average difference of 20.4%, and a medium-sized *d*.

Similar to the nitrate + nitrite concentrations, the orthophosphate (PO_4_^3−^) concentrations in the control treatment were higher in spring (0.55 ± 0.07 µM and 0.17 ± 0.01 µM, respectively) than in fall. The concentrations were negatively affected by warming throughout the spring experiment, with an average decrease of 9.3%. However, the largest negative effect of experimental warming was observed at the end of fall, with an average decrease of 16.7%, between d15 and d17.

Contrary to the nitrate + nitrite and orthophosphate concentrations, the silicate (SiO_2_) concentrations in the control treatments were, on average, lower in spring (3.31 ± 0.18 µM and 10.42 ± 0.15 µM, respectively) than in fall. Warming had a significant positive effect during the second part of the spring experiment (from d10 to d17), with average concentrations being 27.8% higher in the warmed mesocosms, than in the control. In contrast, the strongest effect of warming on silicate concentrations was observed at the end of the fall experiment, when the silicate concentrations were significantly lower in the warmed mesocosms, by an average of 10.8%, between d15 and d17.

The N/P ratio, calculated as the sum of nitrate, nitrite and ammonium concentrations divided by orthophosphate concentration, was 1.99 and 2.39 on average in the control treatment of the spring and fall experiments, respectively (Fig. 1m,n). It was significantly higher over the entire experiments in the warmed treatment by on average 191.5% and 58.8% in spring and fall, respectively (Table 1). In fall, the highest difference between treatments was seen during the second half of the experiment, when the ratio was significantly higher by 133.5% from day 11 to 17.

### Effects of warming on gross primary production and respiration rates derived from oxygen sensor data

**Figure 2:**
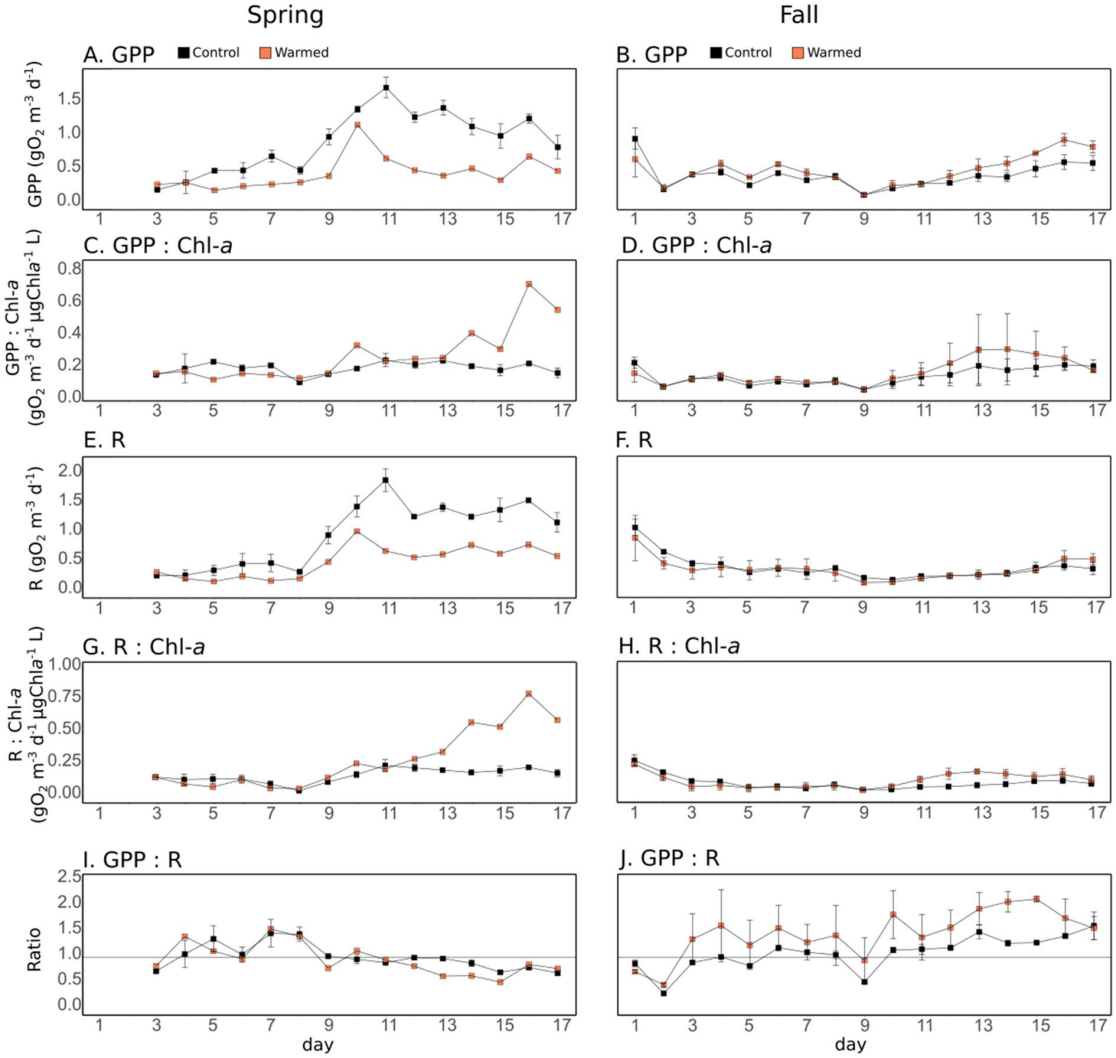
Plankton oxygen metabolism parameters. Gross Primary Production (GPP, **A**, **B**), GPP normalized by the chlorophyll-*a* fluorescence (**C**, **D**), Respiration (R, **E**, **F**), R normalized by the chlorophyll-*a* fluorescence (**G**, **H**), and GPP:R ratio (**I**, **J**) in the control (black) and warmed (orange) treatments. Error bars represent range of observation for the two mesocosms per treatment in spring and the standard deviation for the three mesocosms per treatment in fall. In the spring experiment, GPP: Chl-*a* and R: Chl-*a* could not be estimated on d1 and d2.

In the spring experiment, daily GPP varied between 0.19 ± 0.01 and 1.72 ± 0.15 gO_2_ m^−3^ d^−1^ in the control mesocosms (Fig. 2A). It increased during the first half of the experiment (d2–d10), then decreased toward the end of the experiment. In the fall experiment, the daily GPP was lower than what was observed in the control mesocosms in spring and varied between 0.12 ± 0.02 and 0.96 ± 0.16 gO_2_ m^−3^ d^−1^ (Fig. 2B). In spring, warming significantly reduced GPP by 50.9% over the entire experiment, while in fall, warming enhanced GPP by 21.1% over the entire experiment, 32.3% from d4 to d7, and 44.1% from d12 to d17 (Table 2). In spring, when GPP was normalized by the chl-*a* measured by the high-frequency sensors, it was not significantly different between the treatments, over the entire experiment (Fig. 2C, Table 2). However, it was significantly higher (138%) in the warmed treatment, during the second half of the experiment (d10– d17). In fall, GPP normalized by the chl-*a* was also significantly enhanced (12%) by warming (Fig. 2D, Table 2).

**Table 2:** Summary table of the *p* values and the F-values obtained with the RM-ANOVA (with treatment as fixed factor and time as random factor) comparing the chl-*a* fluorescence, µ, *l*, the µ:*l* ratio, and pigment concentrations in the warmed and in the control treatments over the entire spring and fall experiments or over specific periods defined after trends observed in the data.

Experiment	Variable	Period	*P* value	Cohen’s *d*
Spring	GPP	3–17	**< 1** × **10**^**−4**^ (F_1,14_ = 35.1)	− **1.26**
	GPP: chl-*a*	3–17	0.14 (F_1,13_ = 2.42)	0.67
		10–17	**9.2** × **10**^**−3**^ (F_1,8_ = 14.3)	**1.57**
	R	3–17	**< 1** × **10**^**−4**^ (F_1,14_ = 55.5)	− **1.07**
	R: chl-*a*	3–17	0.07 (F_1,13_ = 3.84)	0.75
		10–17	**4.0** × **10**^**−3**^ (KW)	**1.82**
	GPP: R	3–17	0.39 (F_1,14_ = 0.78)	− 0.16
	chl-*a*	2–17	**< 1** × **10**^**−4**^ (F_1,17_ = 29.4)	− **1.84**
		5–17	**< 1** × **10**^**−4**^ (F_1,17_ = 51.5)	− **3.03**
	µ	2–17	0.17 (F_1,14_ = 2.1)	0.41
		2–7	0.2 (KW)	− 0.49
		8–17	**2.3** × **10**^**−3**^ (F_1,8_ = 19.3)	**1.22**
	*l*	2–17	**0.01** (F_1,14_ = 8.3)	**0.92**
		8–17	**4.1** × **10**^**−3**^ (KW)	**1.62**
	µ:*l*	2–17	0.19 (F_1,14_ = 1.9)	− 0.39
		3–8	**0.046** (F_1,5_ = 6.1)	− **1.46**
	19′-HF	2–18	**4.1** × **10**^**−4**^ (KW)	− **1.45**
	Fucoxanthin	2–18	0.45 (F_1,16_ = 0.59)	− 0.17
	Zeaxanthin	2–18	**0.01** (KW)	− **1.2**
	Chl-*b*	2–18	0.13 (F_1,16_ = 2.5)	0.31
	Peridinin	2–18	**4.3 × 10**^**3**^ (F_1,16_ = 11.2)	**0.87**
	Prasinoxanthin	2–18	0.35 (F_1,16_ = 0.9)	0.16
Fall	GPP	1–17	**0.02** (F_1,16_ = 6.12)	0.41
		4–7	**2** × **10**^**−4**^ (F_1,3_ = 485.4)	**1.28**
		12–17	**2.2** × **10**^**−3**^ (F_1,5_ = 33.4)	**1.22**
	GPP: chl-*a*	1–17	**0.02** (F_1,16_ = 6.79)	0.38
		12–17	**0.03** (F_1,5_ = 9.24)	**1.70**
	R	1–17	0.17 (F_1,16_ = 2.05)	− 0.14
	R: chl-*a*	1–17	0.09 (F_1,16_ = 3.28)	0.35
		11–17	**2.4** × **10**^**−3**^ (F_1,5_ = 32.1)	**3.27**
	GPP: R	1–17	**< 1** × **10**^**−4**^ (F_1,16_ = 36.8)	0.98
	chl-*a*	2–18	0.15 (F_1,15_ = 2.3)	− 0.37
		2–6	**2** × **10**^**−4**^ (F_1,4_ = 163.6)	**1.27**
		8–15	**1** × **10**^**−4**^ (F_1,7_ = 72.1)	− **2.07**
	µ	2–17	**3** × **10**^**−4**^ (F_1,15_ = 22.2)	0.78
		11–17	**2.7** × **10**^**−3**^ (KW)	**3.01**
	*l*	2–17	0.55 (F_1,15_ = 0.38)	0.05
		7–11	**0.01** (F_1,4_ = 32.7)	**1.15**
	µ:*l*	2–17	0.1 (KW)	0.78
		11–17	**1.6 × 10**^**−3**^ (F_1,6_ = 29.9)	**1.55**
	19′-HF	1–18	0.82 (F_1,17_ = 0.05)	0.15
	Fucoxanthin	1–18	**0.01** (KW)	0.72
		13–18	**5.6** × **10**^**−3**^ (F_1,5_ = 21.7)	**1.73**
	Zeaxanthin	1–18	0.52 (KW)	− 0.25
		11–15	**0.01** (F_1,5_ = 19.6)	− **3.35**
		15–18	0.17 (F_1,5_ = 3.1)	**1.54**
	Chl-*b*	1–18	**0.04** (KW)	− **0.85**

Associated Cohen’s *d* effect sizes are also indicated. When the assumptions for a parametric test were not met, despite transforming the data, a Kruskal–Wallis test was used instead and indicated by “KW”. *P* values lower than 0.05 were considered as significant and are indicated in bold in the table. Large and very large Cohen’*s* d effect sizes were also displayed in bold (GPP: Gross Primary Production, chl-*a*: chlorophyll-*a*, R: Respiration, µ: growth rate, *l*: loss rate, 19′-HF: 19′-hexanoyloxyfucoxanthin, Chl-*b*: chlorophyll-*b*).

Daily R varied between 0.27 ± 0.02 and 1.92 ± 0.20 gO_2_ m^−3^ d^−1^ in the spring control mesocosms (Fig. 2E). Similar to the daily GPP, it increased during the first half of the experiment (d2– d10), with a strong increase between d8 and d10, before decreasing slowly until the end of the experiment. In the fall experiment, the daily R was lower than in spring, varying from 0.19 ± 0.02 and 1.09 ± 0.14 gO_2_ m^−3^ d^−1^, in the control mesocosms (Fig. 2F). Warming significantly reduced the daily R by an average of 47.9% in spring, while no significant differences were found in fall (Table 2). During both experiments, when daily R was normalized by chl-*a*, it was not significantly different between treatments over the entire experimental period (Figs. 2G,H), but it was significantly enhanced by warming during the second half of the experiments, by 172% and 49.6%, from d10–17 in spring, and d11–17 in fall, respectively (Table 2).

The GPP:R ratio was on average 1.01 and 1.08 in the spring and fall control treatments, respectively (Fig. 2I,J). Consequently, because warming decreased GPP and R to a similar extent in spring, it did not significantly change the GPP:R ratio. Warming significantly increased GPP:R, by an average of 32% in fall (Table 2).

### Effects of warming on phytoplankton biomass (chlorophyll-*a*), growth, and loss rates derived from the chlorophyll-*a* sensor data

**Figure 3:**
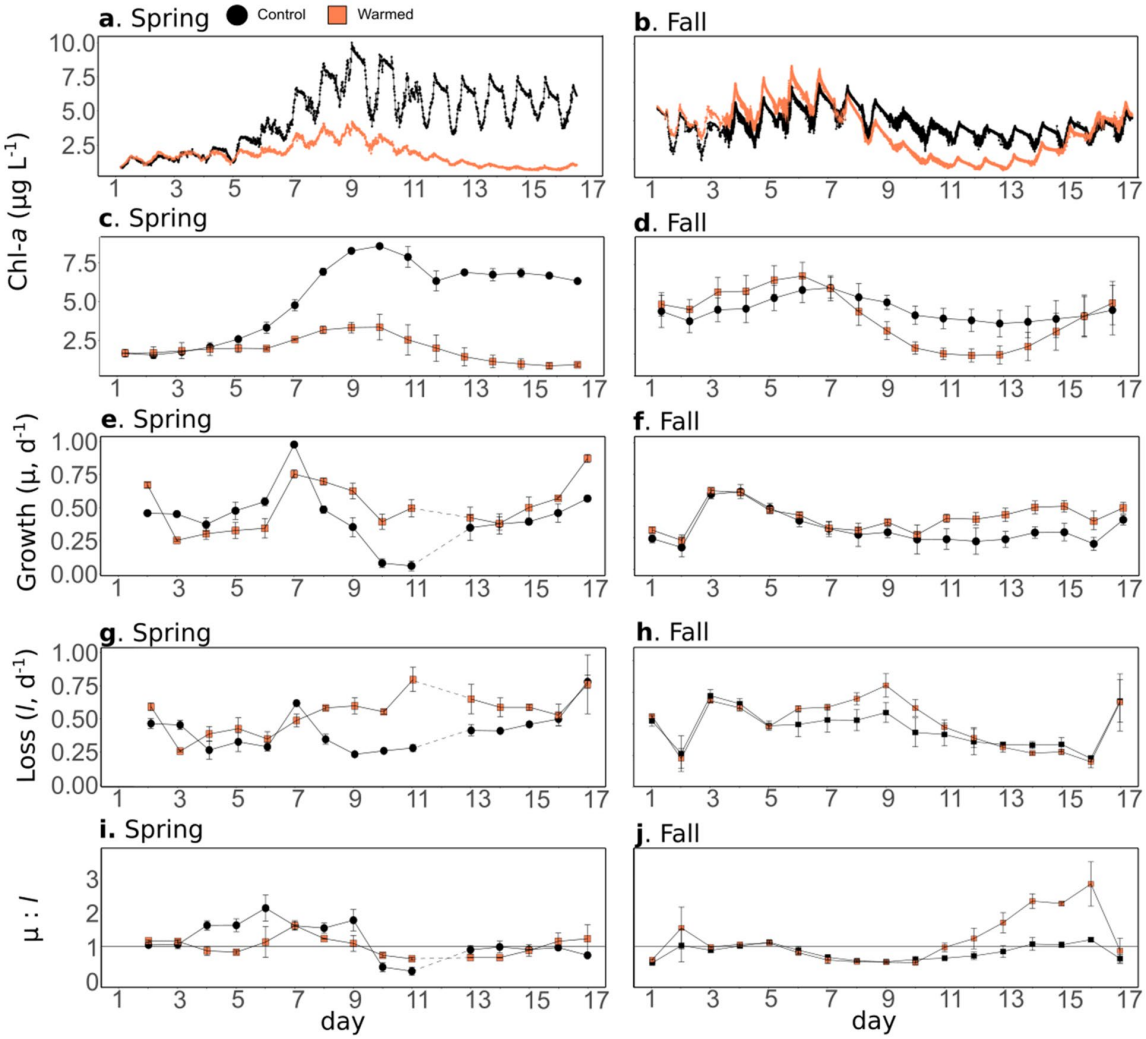
Phytoplankton chlorophyll-*a*, growth and loss rates. High-frequency chlorophyll-*a* data, uncorrected for Non Photochemical Quenching (NPQ) (**a**, **b**), daily average chlorophyll-*a* data corrected for the NPQ(**c**, **d**), phytoplankton growth rate (µ, **e**, **f**), loss rate (*l*, **g**, **h**), and µ:*l* ratio (**i**, **j**) in the control (black) and warmed (orange) treatments. Error bars represent range of observation for the two mesocosms per treatment in spring and the standard deviation for the three mesocosms per treatment in fall. In the spring experiment, µ and *l* could not be estimated on d1 and d12 and, for the latter, the missing data are represented as dotted lines.

The chl-*a* fluorescence data was measured using high-frequency sensors, which were inter-calibrated before and after the experiments, and were corrected by the chl-*a* concentration measured daily by HPLC (see “Methods”). It is hereafter referred to as chl-*a*. In the spring experiment, the daily chl-*a* was 5.28 ± 0.21 µg L^−1^ in the control mesocosms (Fig. 3a,c). A phytoplankton bloom dynamic was observed, with increasing concentrations from d2 to d10, reaching a maximum value of 8.62 ± 0.15 µg L^−1^, and decreasing concentrations from d10 to d17. The average daily chl-*a* was lower in fall than in the spring experiment (4.30 ± 0.59 µg L^−1^) (Fig. 3b,d), and displayed a relatively flat dynamic during the entire experiment, with maximum values on d8 (5.53 ± 0.58 µg L^−1^).

Warming significantly reduced chl-*a* in both experiments (Table 2): an average of 69.5% from d5 to the end of the spring, and 31.7% from d8 to 15, in the fall experiment. Conversely, warming significantly enhanced chl-*a* concentrations at the beginning of the fall experiment (19.4% between d2 and d6). Generally, the magnitude of the effect was larger in spring than in fall (Table 2).

In the control treatment, µ was higher in spring than in fall (0.44 ± 0.04 d^−1^ and 0.32 ± 0.05 d^−1^, respectively; Fig. 3e,f). During both seasons, the maximum µ was observed during the first half of the experiment (spring d7, 0.99 ± 0.01 d^−1^; fall d4, 0.61 ± 0.03 d^−1^). Warming enhanced µ by an average of 18.3% and 28.1%, over the entire spring and fall experiments, respectively, and by an average of 56.8% and 50.9%, respectively, from d8 until the end of the experiment (Table 2). The effect size was higher in fall than in spring (Table 2). However, contrary to the general trend of the entire experiment, during spring, warming significantly reduced µ during the first part of the experiment (d2–d7), with an 18.8% mean difference between the treatments.

In contrast to µ, *l* was almost similar between the seasons, with average values of 0.39 ± 0.04 d^−1^ and 0.40 ± 0.07 d^−1^, in the control treatments for spring and fall, respectively (Fig. 3g,h). In the spring experiment, warming had a positive effect on the mean *l* across the study period (37.1%), and even more from d8 to d17 (59.1%), which was larger than the positive effect found for µ. The effect size of warming was not as large in fall, and *l* was significantly higher in the warmed treatment, although only in the middle of the experiment (20.4% from d7 to d11).

When comparing µ and *l*, the results showed that in spring, µ was higher than *l* in the control treatment, during the first part of the experiment (d2–d9), and lower during the latter half of the experiment (d10–d17). Warming significantly decreased the µ:*l* ratio by 28.9%, during the first half of the experiment (D 3–8, Fig. 3i, Table 2), whereas no significant effect was observed in the rest of the experiment. In the fall control treatment, the µ:*l* ratio was generally lower than that of the spring control (Fig. 3j). Contrary to what was observed in the spring warming, this ratio significantly increased by an average of 92.9%, in the second half of the experiment (d11–d17, Table 2).

### Effects of warming on phytoplankton pigment concentrations

**Figure 4:**
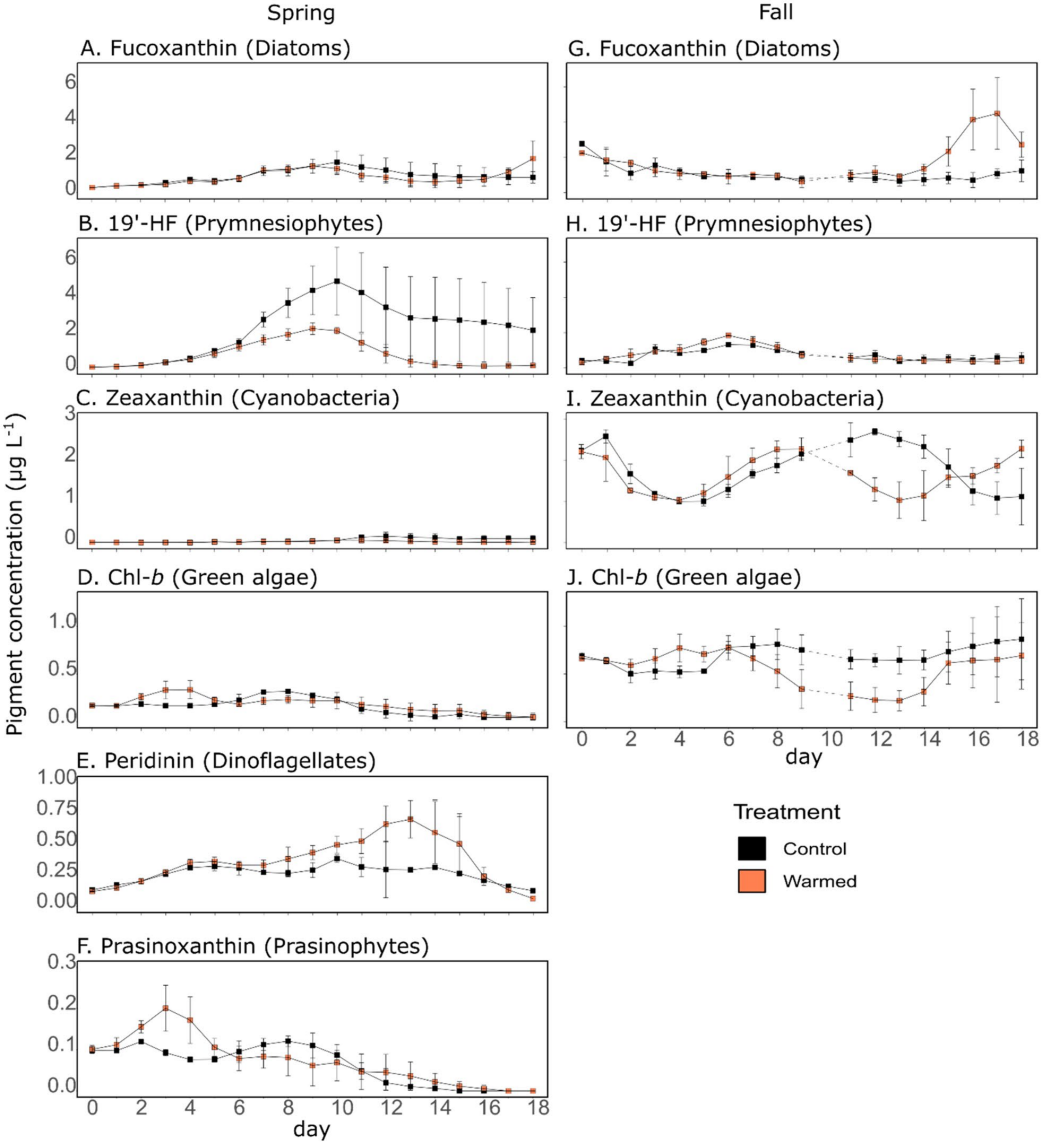
Phytoplankton pigment concentrations. Daily pigment concentrations (µg L^−1^) in the control (black) and warmed (orange) treatments for the spring (**A**–**F**) and fall (**G**–**J**) experiments. Error bars represent range of observation for the two mesocosms per treatment in spring and the standard deviation for the three mesocosms per treatment in fall. Dotted lines represent the missing data on d10 of the fall experiment due to bad weather conditions. (**A**, **G**) fucoxanthin; (**B**, **H**) 19′-hexanoyloxyfucoxanthin; (**C**, **I**) zeaxanthin; (**D**, **J**) chlorophyll-*b*; (**E**) peridinin, and (**F**) prasinoxanthin. Corresponding phytoplankton functional groups are indicated in parentheses.

Phytoplankton pigment composition varied between seasons (Fig. 4). In the spring control treatment, the predominant pigments were fucoxanthin and 19′-hexanoyloxyfucoxanthin (19′-HF), which are mostly associated with diatoms (1.14 ± 0.10 µg L^−1^) and prymnesiophytes (19′-HF, 2.91 ± 0.14 µg L^−1^)^23,24^, respectively (Figs. 4A,B,G,H). The other pigments that were present included peridinin (0.18 ± 0.01 µg L^−1^), Chl-*b* (0.14 ± 0.01 µg L^−1^), zeaxanthin (0.08 ± 0.01 µg L^−1^), and the specific accessory pigment prasinoxanthin (0.06 ± 0.01 µg L^−1^), which are associated with dinoflagellates, green algae, cyanobacteria, and prasinophytes, respectively (Figs. 4C–F,I,J)^23–25^.

In the fall control treatment, the dominant pigments were the cyanobacteria-associated zeaxanthin (1.78 ± 0.22 µg L^−1^), the diatom-associated fucoxanthin (1.07 ± 0.27 µg L^−1^), the green algae-associated Chl-*b* (0.69 ± 0.14 µg L^−1^), and the prymnesiophyte-associated 19′-HF (0.68 ± 0.16 µg L^−1^). Among the main pigments that were identified in the spring experiment, peridinin and prasinoxanthin were either not detected or detected at negligible concentrations in the fall experiment, whereas lutein was detected in fall but not in spring (data not shown).

Warming had seasonal effects on pigment concentrations (Table 2). In the spring experiment, warming had a large and significant negative effect on 19′-HF and zeaxanthin concentrations, with mean concentrations decreasing by 75.4% and 75.2%, respectively. Conversely, warming had moderately significant positive effects on peridinin concentration, which increased by an average of 101%.

In the fall experiment, warming had a significant negative effect on Chl-*b* concentration, which decreased by 19.5%, and on zeaxanthin concentration, which significantly decreased in the middle of the experiment (43.4% from d11 to d15). In contrast, a significant positive effect was observed on fucoxanthin concentration, which increased by 210.7%, during the second part of the experiment (between d13 and d17).

### Relationships between plankton processes, pigment concentrations and environmental parameters

**Figure 5:**
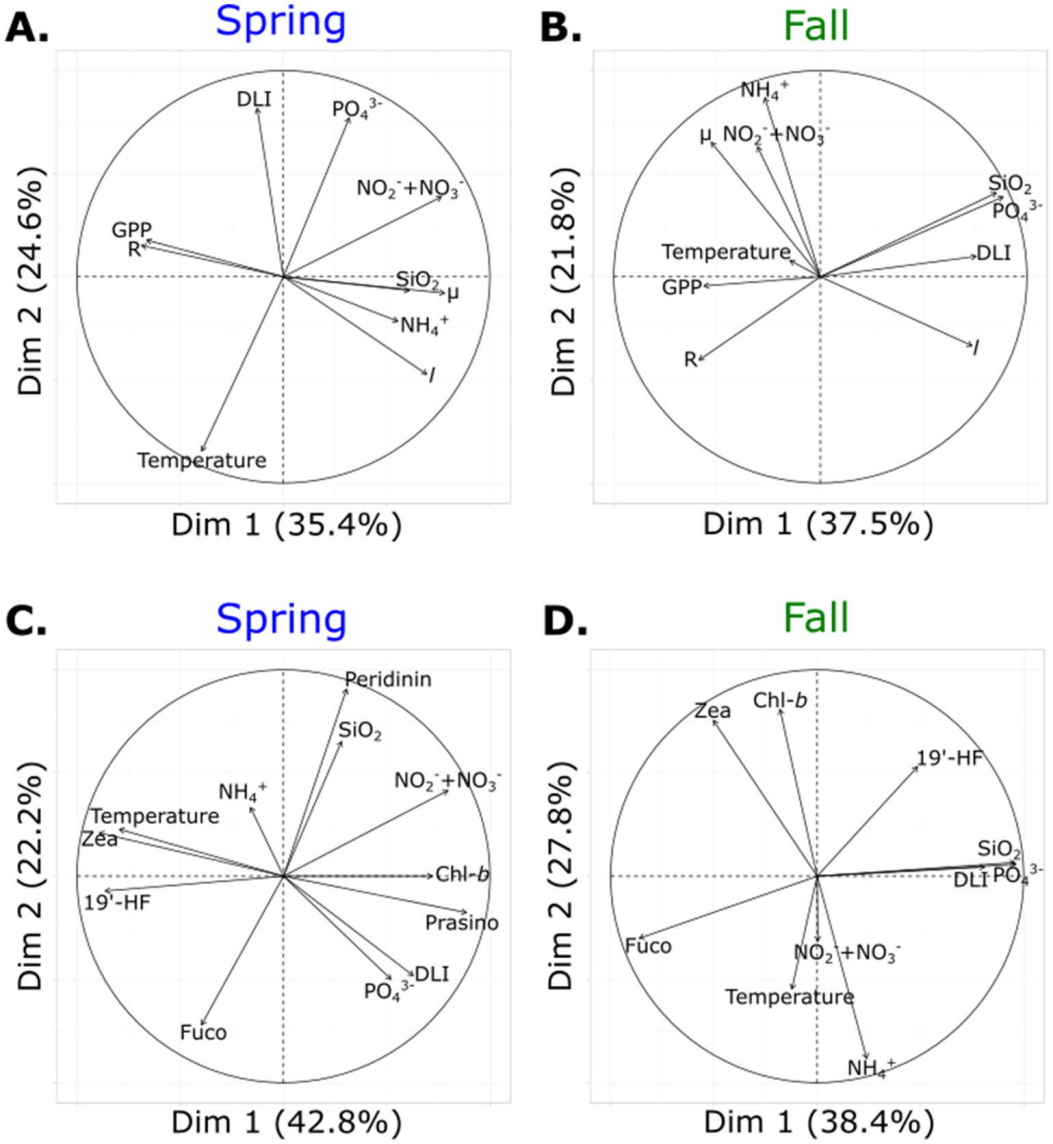
Principal component analyses (PCA) of logarithm response ratio (LRR) of plankton processes (**A**, **B**) and pigment concentrations (**C**, **D**) with environmental parameters for the spring (**A**, **C**) and fall (**B**, **D**) experiments. GPP: Gross Primary Production, R: Respiration, µ: Phytoplankton growth rate, *l*: Phytoplankton loss rate, Chl-*b*: Chlorophyll-*b*, 19′-HF: 19′-Hexanoyloxyfucoxanthin, Fuco: Fucoxanthin, Prasino: Prasinoxanthin, Zea: Zeaxanthin, DLI: Daily Light Integral.

Principal component analyses (PCA) were used to project plankton processes, pigment concentrations and environmental parameters in a multidimensional space in order to illustrate relationships among variables in both experiments (Fig. 5). For both experiments, GPP and R were clustered together along the first PCA axis, although they appeared closer in spring than in fall (Fig. 5A,B). Conversely, µ was close to ammonium for both experiments, to silicate in spring and to nitrate and nitrite in fall; and *l* was part of this cluster in spring but not in fall. Concerning phytoplankton pigment composition, in spring, zeaxanthin, associated with cyanobacteria, and 19′-HF, associated with prymnesiophytes, were part of a group together with temperature (Fig. 5C). Similarly, prasinoxanthin, associated with prasinophytes, and Chl-*b*, associated with green algae, were grouped with DLI and orthophosphate. Finally, peridinin, associated with dinoflagellates, and silicate were clustered together and opposed to fucoxanthin, which is associated with diatoms. In fall, zeaxanthin and Chl-*b*, representing cyanobacteria and green algae, were part of a group opposed to N-nutrients and temperature, while fucoxanthin was opposed to DLI, silicate and orthophosphate (Fig. 5D).

**Figure 6:**
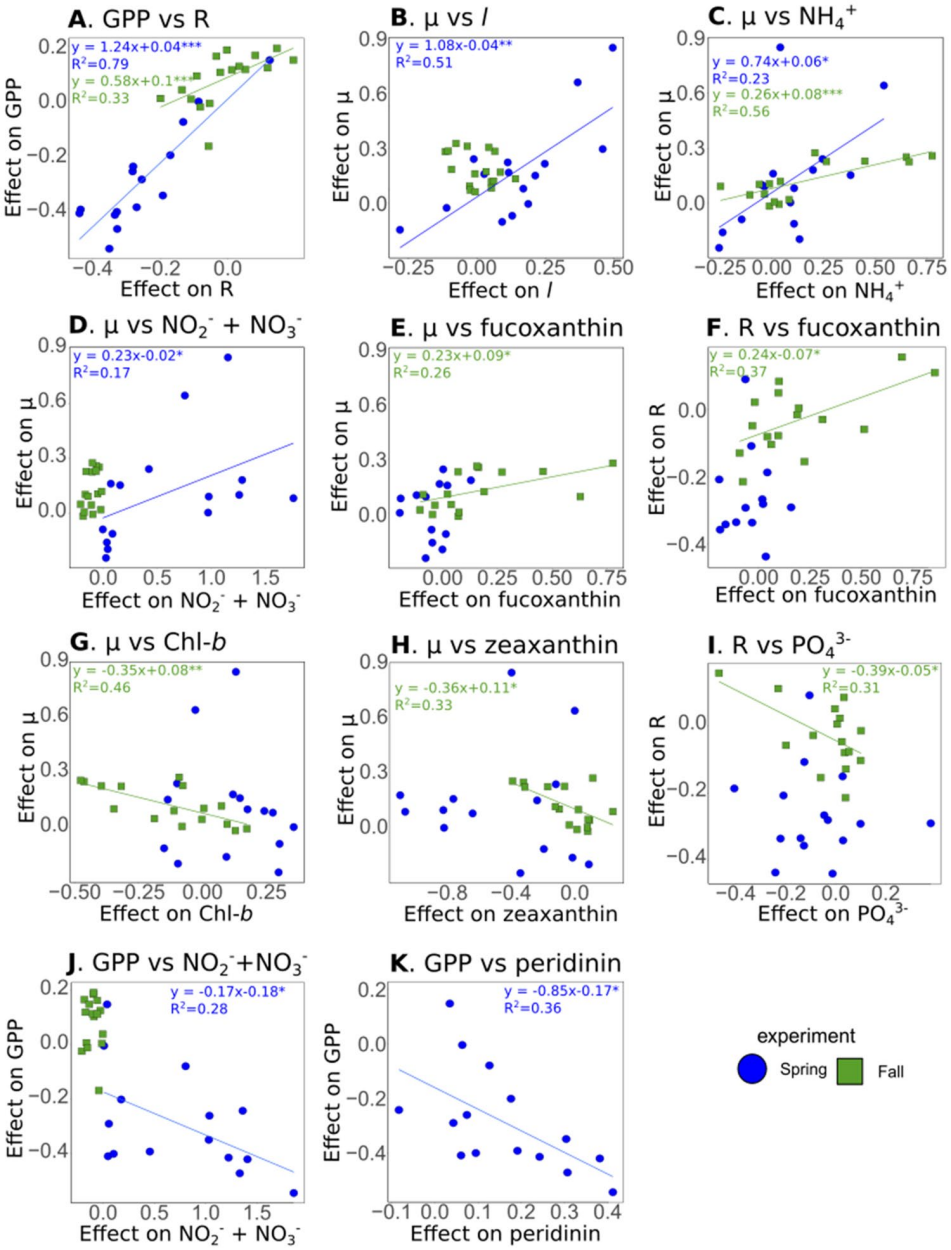
Linear relationships between the effect of warming on plankton processes, environment variables and pigment concentrations. Ordinary least squares linear relationships between the effect of warming, expressed as the log response ratio, on GPP, R, µ, and *l*, and the effect of warming on environmental and pigment variables for the spring (blue circles) and fall (green squares) experiments. Relationships were individually assessed for each experiment. Only statistically significant relationships (*p* < 0.05) are represented and solid blue lines represent the linear least square fit for the spring experiment while solid green lines represent the linear least square fit for the fall experiment. (**A**) Gross Primary Production (GPP) vs Respiration (R), (**B**) growth rate (µ) vs loss rate (*l*), (**C**) µ vs NH_4_^+^, (**D**) µ vs NO_2_^−^ + NO_3_^−^, (**E**) µ vs fucoxanthin, (**F**) R vs fucoxanthin, (**G**) µ vs chlorophyll-*b* (Chl-*b*), (**H**) µ vs zeaxanthin, (**I**) R vs PO_4_^3−^, (**J**) GPP vs NO_2_^−^ + NO_3_^−^, (**K**) GPP vs peridinin.

To evaluate specific relationships between phytoplankton processes, environmental variables, and phytoplankton community composition, ordinary least squares linear relationships were assessed for the effects of warming (expressed as the logarithmic response ratio) on GPP, R, µ, and *l*, nutrient concentrations, DLI, and pigment concentrations (Fig. 6). A significant positive relationship was found between the effects of warming on GPP versus R, and µ versus *l* (Fig. 6A,B). Moreover, the effect of warming on µ was positively and linearly related to the effects of warming on ammonium in both seasons (Fig. 6C), and to nitrate + nitrite concentrations in spring (Fig. 6D). There was no relationship between the effects of µ on pigment concentrations in the spring experiment, but its effects were positively correlated with the diatom-associated pigment fucoxanthin in fall (Fig. 6E). Similarly, R was positively correlated with fucoxanthin in fall (Fig. 6F). In contrast, significant negative relationships were found between the effects of warming on µ, Chl-*b*, and zeaxanthin, the pigments associated with green algae and cyanobacteria, respectively (Fig. 6G,H). Similarly, the effect of warming on R was negatively correlated to orthophosphate (Fig. 6I), and the effect on GPP to nitrate + nitrite and peridinin concentrations (Fig. 6J,K).

### Cumulative GPP, R, and chl-*a*

In the control treatment, the cumulative GPP and R from d2 to d17 (i.e., when experimental warming was fully achieved) were higher in spring than in fall (Table 3). In spring, warming reduced the cumulative GPP and R by 50% and 47%, respectively, while in fall, warming increased the cumulative GPP by 30% and reduced the cumulative R by 5%. Consequently, in the warmed treatment, the cumulative GPP from d2 to d17 was lower in spring than in fall, and conversely to cumulative R, which was higher in spring than in fall. Similarly, in the control treatment, the cumulative chl-*a* was higher in spring than in fall. Warming reduced it by 65% in spring and increased it by 9% in fall. Consequently, in the warmed treatment, cumulative chl-*a* was lower in spring than in fall, whereas it was the opposite in the control treatment. Finally, when combining the spring and fall data, the cumulative GPP, R, and chl-*a* were 26%, 35%, and 32% lower, respectively, under warming.

**Table 3:** Cumulative GPP, R, and chl-*a* from d2 to d17 in the control and the warmed treatment for the spring experiment, the fall experiment, and when adding both experiments, and relative difference between treatments.

Experiment	Parameter	Cumulative value in the control from d2 to d17	Cumulative value in the warmed treatment from d2 to d17	% difference
Spring	GPP	13.95 ± 1.01 gO_2_ m^−3^	7.02 gO_2_ m^−3^	− 50
	R	14.97 ± 0.92 gO_2_ m^−3^	7.93 gO_2_ m^−3^	− 47
	chl-*a*	83.29 ± 2.17 µg L^−1^	29.30 ± 0.93 µg L^−1^	− 65
Fall	GPP	5.93 ± 0.74 gO_2_ m^−3^	7.69 ± 0.99 gO_2_ m^−3^	30
	R	5.63 ± 0.63 gO_2_ m^−3^	5.36 ± 0.86 gO_2_ m^−3^	− 5
	chl-*a*	66.14 ± 1.49 µg L^−1^	71.75 ± 1.30 µg L^−1^	9
Spring + fall	GPP	19.88 gO_2_ m^−3^	14,71 gO_2_ m^−3^	− 26
	R	20.60 gO_2_ m^−3^	13.29 gO_2_ m^−3^	− 35
	chl-*a*	149.43 µg L^−1^	101.05 µg L^−1^	− 32

*GPP* gross primary production, *R* respiration, *Chl-a* chlorophyll-*a.*

## Discussion

There is increasing evidence that the response of plankton to warming is often complex and depends on multiple factors^7,8,11^. Based on high-frequency sensor measurements in the present study, the observed response to warming was relatively similar, in terms of phytoplankton growth and loss rates, in both spring and fall, even though environmental conditions of the system varied between the seasons. In contrast, this was not the case for oxygen primary production and respiration, as warming decreased both GPP and R in spring, whereas GPP was enhanced and no significant effects were observed on R in fall.

The positive effects of warming on µ during both seasons, and GPP in fall, concur with the theoretical effects of warming on metabolic rates^26^. The positive effects of warming, as previously reported, are also in line with global observations, reporting higher GPP and µ under warmer conditions in various regions^27,28^. This is also in accordance with previous mesocosm experiments in coastal waters, which reported high GPP and µ values under experimental warming scenarios^8,29^. Among the environmental factors that can constrain the phytoplankton response to warming, nutrients seem to be the main drivers of growth rate response. In this study, PCAs and linear relationships revealed that only the ammonium, nitrate, and nitrite concentrations showed a positive relationship to µ, while there was no relationship found between daily light radiation and water temperatures with phytoplankton growth rates. More specifically, the positive effect of warming (expressed as the logarithmic response ratio) on ammonium concentrations was linearly related to the positive effect on µ, in both experiments, whereas this was found for nitrate and nitrite concentrations only in spring. These results suggest nitrogen availability as a key parameter in regulating phytoplankton growth response to warming in both seasons, which corresponds with previous studies that found nitrogen to be a limiting factor in the Thau Lagoon^30,31^, and that resource availability generally offset the effects of warming^32^. During both experiments, nitrogen availability was in the range of what was previously reported in the lagoon in spring^6^ and fall^7^, suggesting that future warming may have a greater effect on µ in spring than in fall due to higher nutrient availability, as reported in the present study. In this regard, a change in the lagoon trophic status toward oligotrophication, as currently observed^33^, could mitigate the effect of warming on phytoplankton growth in the future. Similarly, the negative effect of warming on µ reported at the beginning of the spring experiment was probably due to low orthophosphate concentrations, which could have exacerbated competition with bacteria in the warmed mesocosms^34^. Furthermore, the ratio between dissolved inorganic nitrogen and dissolved inorganic phosphorus, which can be a major regulator for growth of certain phytoplankton species^35^, may explain the differences reported in phytoplankton response to warming in spring and fall as this ratio was significantly and strongly enhanced under warming during the entire spring experiment, while it was mainly the case only during the second half of the fall experiment, suggesting a quicker depletion of phosphorus in spring than in fall. In addition, it should be noted that dissolved organic nitrogen and phosphorus concentrations, which were not measured during the present study, could also have played a role in regulating phytoplankton response to warming, as some phytoplankton species mainly access P and N from their dissolved organic pool^36^. Moreover, they might have modified interactions between phytoplankton and bacteria, as they also play an important role in bacteria metabolism^37^.

On the contrary, the negative effect of warming on GPP and R in spring, and the absence of an effect on R in fall, were unexpected, as they contradicted known theoretical effects of warming on metabolism. However, when normalized by chl-*a*, both GPP and R displayed a positive effect of warming in spring, in line with the theory. Similarly, a positive effect of warming was observed on R during the fall experiment when it was normalized by chl-*a*. These results indicate that the negative effects of warming on GPP and R in spring were likely due to the strong decrease in phytoplankton biomass under warming. As both GPP and R decreased by the same magnitude (more or less by half) under warming in spring, this further suggests a tight coupling between phytoplankton and bacteria, as GPP is carried out by phytoplankton, whereas community R is generally dominated by bacterial respiration in coastal waters^38^. The multivariate analysis confirmed this coupling, which appeared stronger in spring than in fall. This difference might be explained by the stronger effect of warming on phytoplankton biomass in spring than in fall, which consequently affected more strongly heterotrophs, notably bacteria that had less phytoplankton derived organic matter available for their metabolism. Overall, these results indicate that plankton oxygen metabolism is strongly driven by the fate of phytoplankton biomass. This highlights the importance of assessing the fate of phytoplankton through measurements of growth and loss, when testing for the effects of warming on the functioning of plankton communities.

As both GPP and R were strongly depressed, to a similar extent in spring, warming did not have significant consequences on the GPP:R ratio. This suggests that warming did not change the capacity of Thau Lagoon to act as a net oxygen consumer in spring. Nevertheless, both cumulative GPP and R (over the entire experimental period when warming was achieved) were strongly depressed by almost half under warming in spring, indicating that oxygen fluxes were significantly altered by warming. In fall, the situation is completely different as warming switched the metabolic status of the system toward autotrophy, shifting it from an oxygen sink to an oxygen net producer. As diatoms generally contribute greatly to primary production in Thau lagoon^39^, and as they were strongly depressed by warming in spring while they were favored at the end of the fall experiment, the difference between spring and fall primary production response to warming might be mostly related to diatoms. Furthermore, this increase in oxygen production attenuated the strong decrease in oxygen production induced by spring warming. However, this mitigation was only partial, as cumulative GPP was reduced by 26% under warming when the spring and fall experiments were cumulated. Nevertheless, our study is the first to show that warming made a fall community as productive as a spring one in a coastal Mediterranean lagoon in terms of oxygen production, in contradiction to what occurs in the lagoon currently.

In addition to enhancing µ during both experiments, warming also significantly increased *l*, which corresponds with the findings of a previous experimental study, which showed that warming increased the grazing activity of microzooplankton on phytoplankton during a spring bloom^40^. Similarly, a global analysis also showed the positive effect of warming on microzooplankton grazing rates under eutrophic conditions^27^. In the present study, the magnitude of the warming effect on *l* varied between seasons; *l* increased considerably throughout the spring experiment and, although to a lesser extent, in the middle of the fall experiment. In natural environments, *l* can be due to grazing by predators, viral lysis, natural death, and sedimentation^18,41^. Warming can promote the grazing of phytoplankton by increasing predator metabolism^10,40^ and enhances viral abundance, thus potentially increasing viral lysis^7,42^. Hence, the comparatively lower effect of warming on *l* in fall might indicate a lower effect of warming on the grazer communities, potentially due to a higher temperature tolerance of grazers at this season, possibly because of differences in zooplankton community composition and/or abundances and/or activity. It could also be the consequence of multiple cascading effects and complex interactions among grazers. For example, Lewandowska et al.^11^ found that zooplankton reduced the grazing pressure on phytoplankton by switching to ciliates under warming and low-nutrient conditions. This could have potentially occurred in fall, as nutrient concentrations, except for silicate, were lower than those in spring. The lower effect of warming on *l* in fall compared to spring could also indicate lower viral lysis, which is consistent with lower viral abundances reported in the lagoon in fall than in spring^6,7^.

In natural environments, the µ:*l* ratio drives phytoplankton dynamics^18^. Warming has been suggested to increase this ratio at low chl-*a* levels and decrease it at high chl-*a* levels^27^. In the present study, even though the average chl-*a* level was similar between the control mesocosms in both experiments, warming considerably reduced the µ:*l* ratio in spring and increased it in fall. Therefore, the results of these experiments suggest that warming could lead to an overall decrease in phytoplankton biomass stock during the spring blooms (− 50%), and an increase during fall (+ 21%). Indeed, warming has been reported to decrease phytoplankton biomass, notably during spring blooms in the Northern Hemisphere^43,44^. However, in contrast, the effects of warming on µ and *l* in fall, when compared to spring, are not well documented in the literature. The present study is the first to document the effects of warming promoting µ more than *l* in a Mediterranean coastal lagoon phytoplankton community in fall. This is of interest for coastal waters in fall as it resulted in an overall increase of phytoplankton biomass which could partly mitigate the strong negative effects of warming on phytoplankton biomass as reported during the spring bloom.

As previously discussed, there are questions as to whether the response of phytoplankton communities depends on environmental status (i.e., nutrient conditions), or on community composition. It is important to note that the results presented herein consider two different phytoplankton communities. Among the different communities observed, in the two experiments, the functional pigment groups showed contrasting patterns. For example, the diatoms and prymnesiophytes were negatively affected by warming in spring, and were either positively affected (diatoms) or unaffected (prymnesiophytes) in fall. Similarly, green flagellates, while being not significantly affected in spring, were suppressed in fall. Hence, the functional pigment groups responded differently according to the experiment; however, these results suggest that warming depressed the functional pigment groups which dominated the phytoplankton community at the beginning of the experiment, i.e., the diatoms and prymnesiophytes in spring, and the green flagellates in fall. Moreover, no specific phytoplankton group was positively correlated with µ in spring, suggesting that the µ response to warming was a collective response, rather than group-specific. In contrast, diatoms were the main contributors to the µ response to warming in fall. This is validated by previous studies, which similarly reported diatoms as major components of phytoplankton assemblages, contributing greatly to µ, under natural in situ conditions^39,45^.

Describing planktonic process responses to relevant disturbances could prove useful in predicting the fate of plankton communities and the overall primary productivity of our oceans under future climate scenarios. The present study highlights the potential of assessing GPP, R, µ, and *l* responses to warming with automated high-frequency sensors, which provide continuous data, regardless of the accessibility of the mesocosms. In conclusion, the use of high-frequency sensors immersed in in situ mesocosms allowed us to investigate the changes induced by warming on key planktonic processes and to further elucidate the complex effects of warming on coastal Mediterranean assemblages during two contrasting, productive seasons. Warming affected the fate of phytoplankton and the metabolic status of the lagoon during the two seasons, and systematically depressed the phytoplankton groups that dominated the community at the start of the experiments. Overall, warming shifted the production balance of the Thau Lagoon towards fall, by strongly depressing the phytoplankton biomass and oxygen production during the spring bloom and enhancing it in fall. This resulted in the fall community being as productive as the spring one under warming. Our experiments indicate that the strong negative effects of warming on phytoplankton biomass and production that occurred in spring were only partially mitigated by their increase in fall. When the two experiments were combined, the overall effects of warming were negative for oxygen production, respiration, and phytoplankton biomass. By highlighting the contrasting effects of the same disturbance performed at the same location during two different seasons, this study emphasizes the importance of conducting studies at relevant spatial and temporal scales, to obtain a more comprehensive view of a community-wide response to a given disturbance. It suggests that future global warming could potentially weaken spring production in otherwise productive coastal waters, such as in the Thau Lagoon. Conversely, it can enhance fall production without full recovery of the lost spring production.

## Methods

### In situ mesocosm experiments

Two in situ mesocosm experiments were conducted in 2018 in the Mediterranean coastal Thau Lagoon at the Mediterranean platform for Marine Ecosystem Experimental Research facility (MEDIMEER: 43°24′53″ N, 3°41′16″ E). The spring experiment was performed for 18 days in April 2018, and the fall experiment was performed for 18 days in October 2018. The mesocosms consisted of 280 cm high and 120 cm wide cylindrical bags made of 200-µm-thick transparent vinyl acetate polyethylene film reinforced with nylon (Insinööritoimisto Haikonen Ky). They were covered with a transparent dome made of polyvinyl chloride and moored individually to a floating pontoon. All mesocosms were simultaneously filled with 2200 L of lagoon water screened through a 1000-µM mesh to remove large particles and organisms, resulting in a mesocosm water column of approximately 2 m. A more precise description of the mesocosm filling procedure is provided in Courboulès et al.^7^. In order to prevent stratification, a pump (Rule, Model 360) to gently mix the water column was immersed at 1 m depth, resulting in a turn-over rate of approximatively 3.5 d^−1^. All methods were performed in accordance with the relevant guidelines and regulations.

The mesocosms were equipped with a set of automated sensors immersed at a depth of 1 m and programmed for high frequency recording (one measurement every 15 min for the spring experiment, and one measurement every 1 min for the fall experiment). Each set was composed of a fluorometer (ECO-FLNTU, Wetlabs) for chl-*a* fluorescence, oxygen optode (3835, Aanderaa) for dissolved oxygen (DO) concentration, electromagnetic induction conductivity sensor (4319, Aanderaa) for conductivity, spherical underwater quantum sensor (Li-193, Li-Cor) for photosynthetically active radiation (PAR), and three temperature probes (Thermistor probe 107, Campbell Scientific) for water temperature at three depths (0.5, 1, and 1.5 m).

### Water temperature control in the mesocosms

In the spring experiment, three mesocosms served as controls and were maintained at a similar temperature as the lagoon and three other mesocosms were warmed at + 3 °C compared to the in situ lagoon temperature (hereafter referred to as C1-Spring, C2-Spring and T1-Spring, T2-Spring, respectively), Only two of each type of mesocosm were equipped with sensors and used for the present study. In the fall experiment, three mesocosms were used as controls and equipped with sensors (hereafter referred to as C1-Fall, C2-Fall, and C3-Fall) and three mesocosms were warmed at + 3 °C and equipped with sensors (T1-Fall, T2-Fall, and T3-Fall). Hence, data were represented as mean ± range of observations for the spring experiment and as mean ± standard deviation from the mean for the fall experiment. Heating was adjusted in real-time to follow the natural temperature variations of the lagoon, as described in previous studies^7,8,46^.

### Nutrient analysis

The dissolved nutrient concentrations were measured daily using the same protocol in both experiments. Water was sampled from each mesocosm at a depth of 1 m every morning using a 5 L Niskin water sampler. Samples (50 mL) from the Niskin water sampler were placed in acid-washed polycarbonate bottles and filtered with 0.45 µm filters (Gelman) using a low-vacuum pump, before being stored at − 20 °C. Dissolved nutrient (ammonium [NH_4_^+^], nitrate [NO_3_^−^], nitrite [NO_2_^−^], orthophosphate [PO_4_^3−^], and silicate [SiO_2_]) concentrations were determined using a colorimeter (Skalar Analytical) and following the protocol detailed in Hydes et al.^47^.

### Phytoplankton pigment composition

Phytoplankton pigment composition was assessed daily in both seasons. Samples were taken from each mesocosm at a depth of 1 m every morning using a 5 L Niskin water sampler. Each sample (800–1500 mL) was filtered at low ambient light on a glass-fiber filter (Whatman GF/F, 25 mm diameter, 0.7 µm nominal pore size) using a low-vacuum pump, and frozen at − 80 °C. Pigment extraction consisted of two steps. First, the filters were placed in 2 mL of 95% ethanol and stored at − 20 °C for 1 h. Then, filters were sonicated and stored at − 4 °C for 1 h. Finally, the extracts were clarified using a glass-fiber filter (Whatman GF/F). The extracts were then directly analyzed using HPLC (Waters), following the method of Zapata et al.^48^ and the detailed protocol of Vidussi et al.^8^.

Some pigments can be used as taxonomic biomarkers because they are representative of particular phytoplankton groups^23–25^. Accordingly, chlorophyll-*b* (chl-*b*) was attributed to green algae, prasinoxanthin to prasinophytes, fucoxanthin to diatoms, zeaxanthin to cyanobacteria, 19′-HF to prymnesiophytes, and peridinin to dinoflagellates. Ubiquitous pigments, being present in multiple groups, were not assigned a specific phytoplankton group.

### High-frequency sensor data acquisition, calibration, and correction

Raw fluorescence data were systematically transformed into µg chl-*a* L^−1^ (hereafter referred to as µg·L^−1^) following the manufacturer’s recommendations. Each chl-*a* fluorometer was calibrated using an algae monoculture for which the chl-*a* concentrations were measured using high-performance liquid chromatography (HPLC). For the spring experiment, calibration was performed with *Dunaliella tertiolecta* (Dunaliellaceae), with six chl-*a* concentration points ranging from 0 to 10 µg L^−1^. For the fall experiment, the fluorometers were calibrated with a culture of *Tetraselmis chui* (Chlorodendraceae) and with five chl-*a* concentration points, from 0 to 12.7 µg L^−1^. After the data were corrected with calibration coefficients, they were additionally corrected using the daily chl-*a* concentration measured by HPLC from samples taken in every mesocosm once a day at 09:00. Finally, the chl-*a* sensor data were corrected for non-photochemical quenching (NPQ), as a strong decrease in chl-*a* occurred in almost all daily chl-*a* cycles during sunlight. To account for NPQ, chl-*a* data were linearly interpolated between sunrise and sunset^49,50^. This interpolation assumed that spatial homogeneity in the phytoplankton community inside each mesocosm was achieved with the use of a pump to gently mix the water column^51^.

Each oxygen optode was calibrated before and after the experiments with three saturation points (0, 50 and 100%) at different temperature levels (17–22 °C). The 0 and 50% saturation points were obtained by adding potassium metabisulfite into distilled water, while the 100% saturation point was reached by bubbling air into the distilled water. The dissolved oxygen data obtained during the experiments were also corrected for salinity and temperature measured with the conductivity and the temperature sensors immersed in the mesocosms. The dissolved oxygen data were finally corrected with dissolved oxygen concentrations that were measured with the Winkler method. All the calibration and the correction procedures are detailed in Soulié et al.^21^.

### Daily Light Integral (DLI) from high-frequency PAR measurements

The daily light integral, the quantity of photosynthetically active photons received on a 1 m^2^ surface over a 1 d period^52^, was estimated using high-frequency PAR measurements at 1 m depth, with Eq. (1):

1 $$DLI = \frac{mean\, PAR \times\, day\, length \times 3600}{{1 \times 10^{6} }}$$

With the DLI expressed in mol m^−2^ d^−1^, mean PAR between sunrise and sunset as µmol m^−2^ s^−1^, and day length in h being the duration between sunrise and sunset.

### Phytoplankton growth and loss rate estimations using high-frequency sensor data

Phytoplankton µ and *l* were estimated using the corrected chl-*a* fluorescence data and a method detailed in Soulié et al.^50^ and following similar principles as in Neveux et al.^53,54^. The daily chl-*a* cycle was separated into two parts: the “increasing period,” which starts from sunrise until the fluorescence maximum is reached (generally a few hours after sunset), and the “decreasing period,” from the time of maximum fluorescence until the following sunrise. The maximum chl-*a* always occurred several minutes to a few hours after sunset. For each period, an exponential regression was performed using the chl-*a* as y and time as x, following Eq. (2):

2 $$Chla = a \times e^{bt}$$

With $Chla$ the chl-*a* (µg L^−1^), *a* (µg L^−1^) and *b* (min^−1^) constants, and t the time (min). Following Siegel et al.^55^ and Neveux et al.^53,54^, as there is no growth during the night and mesocosms are continuously mixed and enclosed systems, the changes in $Chla$ are assumed to be due to *l* at night during the “decreasing period.” Therefore, during the “decreasing periods,” $b = l$, with *l* in min^−1^, and during the “increasing periods,” $b = \mu - l$. Subsequently, µ (min^−1^) was calculated using Eq. (3):

3 $$\mu = \left( {\mu - m} \right) + l$$

Finally, *l* and µ were converted to h^−1^ by multiplying by 60 to obtain the hourly rates. Then, hourly *l* rates were multiplied by 24 to obtain daily rates (d^−1^). Here, we assumed that losses occurred during the entire 24-h period, and hourly µ rates were multiplied by the duration of the increasing period, in hours, as growth only occurred during the increasing period^52–54^.

### Gross primary production and community respiration estimation using high-frequency sensor data

GPP and R were estimated from high-frequency dissolved oxygen data using a free-water diel oxygen method based on the classical technique from Odum^21,56^. Each dissolved oxygen cycle was separated in periods of positive instantaneous Net Community Production (NCP) and periods of negative instantaneous NCP (periods of increasing and decreasing dissolved oxygen concentration, respectively). For each positive and negative NCP periods, the dissolved oxygen data were smoothed with a 5-point sigmoidal model. The fundamental equation of the method is presented as Eq. (4):

4 $$\frac{{\Delta O_{2} }}{\Delta t} = GPP - R - F - A$$

The instantaneous change in dissolved oxygen $\frac{{\Delta O_{2} }}{\Delta t}$ is considered to depend on GPP, R, and on F, which represents the physical oxygen exchange between the water and the atmosphere, and A, which encompasses all other phenomena which could affect the dissolved oxygen concentration. A was taken as null in the present work as in most other studies^21,50,57^. F was calculated as follows (Eq. 5):

5 $$F = \left( {k \times (O_{2} - O_{2sat} } \right))/Z_{mix}$$

In this equation, k represents the piston velocity coefficient, $O_{2}$ and $O_{2sat}$ the concentration and saturation of dissolved oxygen respectively, and $Z_{mix}$ the water column mixing depth, which is the mesocosm water column length in the case of mixed mesocosms^50,52^. The k value was taken as k = 0.000156 m min^−1^ from the literature^58^. Then, k was corrected for temperature and salinity using the high-frequency sensors data and following the procedure described in Soulié et al.^50^.

Then, instantaneous NCP was calculated from the following equation (Eq. 6):

6 $$NCP\left( t \right) = O_{2} \left( t \right) - O_{2} \left( {t - 1} \right) - F\left( t \right)$$

In this equation, $O_{2} \left( t \right)$ and $O_{2} \left( {t - 1} \right)$ are the dissolved oxygen concentration at time t and t − 1, respectively, and $F\left( t \right)$ the exchange factor at time t. From this instantaneous NCP data, daily metabolic parameters were inferred. First, the respiration occurring during daylight, Rdaytime, was estimated with Eq. (7):

7 $$R_{daytime} = (mean\,of\,NCP\,during\,a\,1h\,period\,centered\,around\,the\,max. \, NCP\,of\,the\,Negative\,NCP\,period) \times duration\,of\,Positive\,NCP\,period \times 60$$

In this equation, Rdaytime is expressed in gO_2_ m^−3^ d^−1^, the mean instantaneous NCP in gO_2_ m^−3^ min^−1^, and the duration of the positive NCP period in h. The respiration occurring at night, Rnight, was estimated from Eq. (8):

8 $$R_{night} = (mean\,of\,NCP\,during\,the\,Negative\,NCP\,period) \times duration\,of\,Negative\,NCP\,period \times 60$$

Similarly, Rnight is expressed in gO_2_ m^−3^ d^−1^, the mean instantaneous NCP in gO_2_ m^−3^ min^−1^, and the duration of the negative NCP period in h. Finally, daily R is the sum of Rdaytime and Rnight.

Daily GPP is then calculated with the following equation Eq. (9):

9 $$GPP = R_{daytime} + (mean\,of\,NCP\,during\,the\,Positive\,NCP\,period) \times duration\,of\,Positive\,NCP\,period \times 60$$

Daily GPP and Rnight are expressed in gO_2_·m^−3^·d^−1^, the mean instantaneous NCP in gO_2_·m^−3^·min^−1^, and the duration of the positive NCP period in h. Daily NCP is then calculated as the difference between daily GPP and daily R.

### Cumulative GPP, R and chl-*a* data

As warming was fully achieved from day 2 onwards, cumulative data from d2 to d17 were calculated for GPP, R, and daily chl-*a*, to compare the overall consequences of warming on both experiments, using Eq. (10):

10 $$\mathop \sum \limits_{i = 1}^{n} x_{i}$$

With $x_{i}$ the value of the investigated parameter on day *i*, and n the last experimental day for which the parameter was estimated (n = 17). For the spring experiment, GPP, R and chl-*a* data were missing on d2 and were linearly extrapolated with data from d3 to d6.

### Statistical analysis

To test the effect of warming on the daily time series of DLI, nutrient concentrations, pigment concentrations, GPP, R, µ and *l*, one-way repeated measures analysis of variance (RM-ANOVA) were performed with treatment as a fixed factor and time as a repeated random factor. *P*-values less than or equal to 0.05 were considered representative of a significant effect of the treatment. RM-ANOVAs were performed on the entire experimental time series as well as on shorter periods to assess specific trends. When the normality and homoscedasticity assumptions of the RM-ANOVA were not met, even after logarithmic, exponential or square root data transformation, a non-parametric Kruskal–Wallis test was performed instead. Cohen’s *d* effect sizes were calculated using the mean values of certain parameters to compare the magnitude of the warming effect between the spring and fall experiments. Cohen’s *d* effect size^59^ was calculated as the difference between the mean values in the warmed and control treatments divided by the pooled standard deviation. The effect of warming was considered very small if Cohen’s *d* was higher than |0.01|, small if it was higher than |0.2|, medium if it was higher than |0.50|, large if it was higher than |0.80|, and very large if it was higher than |1.2|. Moreover, to assess the principal drivers of nutrient concentrations, DLI, pigment concentrations, GPP, R, µ and *l* responses to warming, ordinary least squares linear relationships were assessed using the effect of warming expressed as the log response ratio (LRR). This LRR was calculated as $LRR_{X} = {\text{log}}\left( {\frac{{X_{W} }}{{X_{C} }}} \right)$, with *X*_*W*_ and *X*_*C*_ the values of the considered variable *X* in the warmed and control treatments, respectively. Principal Component Analyses (PCA) were performed between LRRs of environmental parameters (nutrient concentrations, DLI, temperature), pigment concentrations, and plankton processes (GPP, R, µ and *l*). All data management and statistical analyses were performed using R software (R-Project, version 4.0.1).

## Data Availability

The datasets used and/or analysed during the current study are available from the corresponding author on reasonable request.
